# Transcranial Doppler for Early Prediction of Cognitive Impairment after Aneurysmal Subarachnoid Hemorrhage and the Associated Clinical Biomarkers

**DOI:** 10.1155/2020/8874605

**Published:** 2020-11-23

**Authors:** Ahmed Esmael, Tamer Belal, Khaled Eltoukhy

**Affiliations:** Neurology Department, Faculty of Medicine, Mansoura University, Egypt

## Abstract

**Methods:**

Prospective study included 40 cases with acute aSAH. Initial evaluation by Glasgow Coma Scale (GCS) and the severity of aSAH was detected by both the clinical Hunt and Hess and radiological Fisher's grading scales. TCD was done for all patients five times within 10 days measuring the mean flow velocities (MFVs) of cerebral arteries. At the 3-month follow-up, patients were classified into two groups according to Montreal Cognitive Assessment (MoCA) scale: the first group was 31 cases (77.5%) with intact cognitive functions and the other group was 9 cases (22.5%) with impaired cognition.

**Results:**

Patients with impaired cognitive functions showed significantly lower mean GCS (*p* = 0.03), significantly higher mean Hunt and Hess scale grades (*p* = 0.04), significantly higher mean diabetes mellitus (DM) (*p* = 0.03), significantly higher mean systolic blood pressure (SBP) and diastolic blood pressure (DBP) (*p* = 0.02 and *p* = 0.005, respectively), and significantly higher MFVs measured within the first 10 days. The patients with cognitive impairment were accompanied by a higher incidence of hydrocephalus (*p* = 0.01) and a higher incidence of delayed cerebral ischemia (DCI) (*p* < 0.001). Logistic regression analysis detected that MFV ≥ 86  cm/s in the middle cerebral artery (MCA), MFV ≥ 68  cm/s in the anterior cerebral artery (ACA), and MFV ≥ 45  cm/s in the posterior cerebral artery (PCA) were significantly associated with increased risk of cognitive impairment.

**Conclusion:**

Cognitive impairment after the 3-month follow-up phase in aSAH patients was 22.5%. Acute hydrocephalus and DCI are highly associated with poor cognitive function in aSAH. Increased MFV is a strong predictor for poor cognitive function in aSAH. This trial is registered with NCT04329208.

## 1. Introduction

Aneurysmal subarachnoid hemorrhage is a form of cerebral hemorrhage that leads to severe injury to the brain with a significant increase in morbidity and mortality [1–3]. It is a potentially life-threatening disease that is fatal in about 10-25% of all patients with acute SAH [4]. The 6-month mortality rate is up to 50% [5]. Impaired cognitive functions are common neurological manifestations following aSAH [6], even in patients with good neurological outcome [7].

Hütter and colleagues in 1998 determined impaired short and long-term memory, attention, and frontocortical functions in the acute stage following aSAH (average 6 days) [8]. This pattern of cognitive impairment is very comparative to the cognitive dysfunctions found in the chronic stages following aSAH [9–11].

Nordenmark et al. (2019) mention that most studies have depended on the medical variables to predict the cognitive impairment in the chronic stages following aSAH, usually 3 months or 1 year following the onset [12].

The study by Hütter stays the only study of aSAH cases in the acute stage that has associated the medical variables to cognitive impairment and they concluded that the amount of blood (Fisher score), frontal hematoma, intraventricular hemorrhage, and acute hydrocephalus were associated with cognitive impairment in the acute stage [12].

Several researchers found that impaired brain perfusion and cerebral blood flow (CBF) can occur before the onset of the cognitive impairment [13, 14], suggesting that cerebral hypoperfusion is one of the factors by which vascular damage may add to neurodegeneration [15, 16]. In addition, cerebral hypoperfusion was illustrated to be not only an epiphenomenon of loss of the brain tissue but also precipitating, starting, and advancing the neurodegeneration [17, 18].

Early changes in the cerebral blood vessels wall can be dependably distinguished by ultrasound methods, which permit identifying even small changes [19]. Ultrasound can assess structural and functional changes of the cerebral blood vessels that add to the hypoperfusion in cognitive disorders [20]. In specific, TCD is a reasonable, cheap, and portable imaging technique with great reliability [21].

The present study is aimed at predicting the cognitive impairment by TCD and detecting the associated clinical biomarkers of impaired cognition in the aSAH.

## 2. Patients and Methods

### 2.1. Study Design and Participants

A single-center, prospective study was conducted on 102 consecutive patients with acute nontraumatic SAH within 24 hours of the onset diagnosed by clinical examination and the initial brain computed tomography (CT). Patients were admitted to the Neuropsychiatry Department, Mataria Teaching Hospital, over a 1-year period initiated on the 1st of January 2018.

Adult patients (aged 30-65 years) of both sexes presented by spontaneous aneurysmal SAH, diagnosed by noncontrast brain CT scan at the onset, and confirmed by CT angiography within 1-3 days of onset were included.

**Figure 1:**
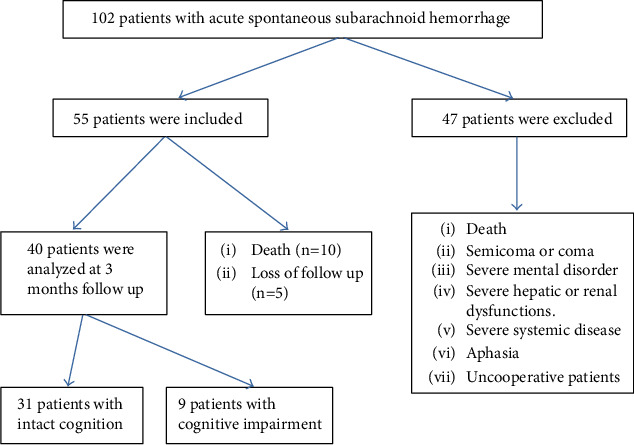
Study flow chart of patients included in the final analysis.

Patients were excluded from this study in the following cases: marked impaired consciousness, marked mental disorders or previous dementia, marked systemic diseases, marked hepatic or renal impairment, aphasia, or noncooperative patients who could not perform the MoCA. Also, patients with previous stroke, other neuropsychiatric disorders, or drug therapy that impairs cognition were excluded. During the follow-up duration after 3 months, 10 cases died and 5 cases missed (Figure 1). Finally, the study involved 40 patients with a mean age of 51.1 ± 13 years consisted of 22 females and 18 males in the final analysis of the results.

### 2.2. Ethical Approval

Written informed consent was provided by all cases or 1^st^-degree relatives. This study was approved by the local Institutional Review Board of Neurology Department, Faculty of Medicine, Mansoura University, Egypt.

### 2.3. Clinical Assessment, Laboratory Investigations, and Radiological Diagnosis of SAH

A complete history was taken including risk factors that may cause aSAH or may impact the cognition such as age, sex, and history of hypertension, DM, hyperlipidemia, and smoking. General examination was carried out with special consideration of systolic and diastolic blood pressures. Also, a complete neurological examination was carried out for all cases included initial GCS score and Hunt and Hess scale grades.

**Figure 2:**
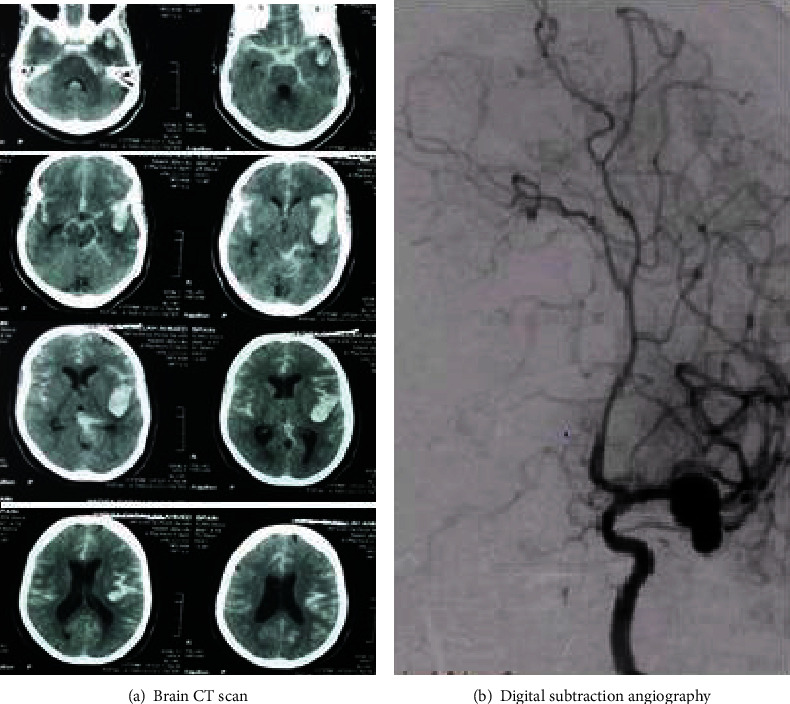
Diagnosis of aSAH by brain CT scan and digital subtraction angiography. (a) Brain CT scan was done revealing subarachnoid hemorrhage. Hunt and Hess scale was III. (b) Digital subtraction angiography was done showing the left MCA aneurysm.

SAH was diagnosed at the onset of admission by noncontrast brain CT scan, and the severity of aSAH was detected by using Fisher's grading scales. Within 1-3 days, the patients were sent for CT angiography and digital subtraction angiography of cerebral arteries to demonstrate the location, number, and size of the ruptured aneurysm (Figure 2). Follow-up brain CT and/or brain MRI scan was done for cases with suspected vasospasm and/or DCI.

Initial laboratory investigations were carried out to all cases after admission including full complete blood picture, random blood glucose, prothrombin time and activity, hepatic function tests, urea and serum creatinine, total lipid profile, and serum electrolytes.

### 2.4. Clinical and TCD Monitoring

Clinical evaluation was carried out throughout the day in the intensive care unit (ICU) and the assessment of the cases by GCS score and Hunt and Hess scale grades. Also, continuous observations of any decrease in the level of consciousness or any focal neurological signs were detected.

TCD examination was performed using DWL-EZ-Dop machine Compumedics GmbH, Singen, Germany. TCD uses low-frequency pulses (2 MHz) for the measurement of MFV in the cerebral arteries determining systolic and diastolic peaks and MFV. MFV is calculated by (systolic + diastolic)/3 + diastolic  velocities, according to Alexandrov et al. [22].

Initial TCD examination was done after admission serving as a baseline state for cerebral circulation. Follow-up TCD examinations were done at fixed intervals on the 1^st^, 3^rd^, 5^th^, 7^th^, and 10^th^ days of the onset of SAH. TCD examination protocol for intracranial arteries was performed according to Alexandrov et al. [22].

Cerebral vasospasm (CV) is defined as a delayed but reversible stenosis of the cerebral blood vessels mostly involving the proximal arteries of the circle of Willis. Clinical vasospasm is narrowing of cerebral artery causing cerebral ischemia, while angiographic vasospasm is stenosis of arteries as demonstrated on vascular imaging [23, 24]. TCD diagnosis of CV is depending on MFV [25]. Vasospasm is considered when the MFV increases over ≥120 cm/s in the MCA, ≥90 cm/s in the ACA, and ≥60 cm/s in the PCA [26–30]. Also, a rapid increase of 50 cm/sec or more during a 24-hr period is a strong predictor of symptomatic CV [31]. Additional investigations as CTA and/or DSA were carried out to confirm the incidence of radiological vasospasm.

### 2.5. Cognitive Assessment at Follow-Up

Cognitive function was assessed by using MoCA score after 3 months of the onset of SAH during the follow-up of patients. Impaired cognitive functions were diagnosed if MoCA scores were less than 26 [32, 33]. According to MoCA scores, patients were classified into two principal groups: the first was cases with intact cognitive functions (MoCA scores more than 25) and the other group was cases with cognitive impairment (MoCA scores less than 26).

#### 2.6. Statistical Analysis

Analyses of data were done by utilizing SPSS version 22. Data are summarized as the mean ± SD and range for continuous variables, and as frequency for categorical variables. Statistical analysis was performed using the *χ*^2^ and Fisher exact tests in categorical variables and the Student *t*-test for comparison of means in continuous variables.

Spearman correlation analysis was carried out between MFV of cerebral arteries and MoCA. *p* value < 0.05 was considered statistically significant.

Odds ratio (OR) was estimated, and the 95% confidence interval (CI) was calculated, and to detect the cut-off values for MFV, a receiver operating characteristic (ROC) curve was done. Lastly, a logistic regression analysis was carried out to estimate the independent predictive ability of the detected cut-off values.

## 3. Results

### 3.1. SAH Patients and MoCA Scale (Table 1)

40 patients with spontaneous SAH were involved in this study with an average age of 51.1 ± 13 years. MoCA scale score after 3 months from the onset of SAH was estimated, and subsequently, cases were classified into two groups: the first included 31 patients with good cognitive functions (77.5%) and the second included 9 patients with poor cognitive functions (22.5%).

The total MoCA scores of the cases of SAH with cognitive impairment were significantly less than cases with intact cognition (23.89 ± 2.46  versus 27.12 ± 2.78, *p* < 0.001). Also, there was a significant impairment in the subtests of MoCA especially the domains of memory, executive functions, naming, and attention, while the domains of the visual-spatial ability, language, and orientation were nonsignificantly diminished after SAH with impaired cognition.

**Figure 3:**
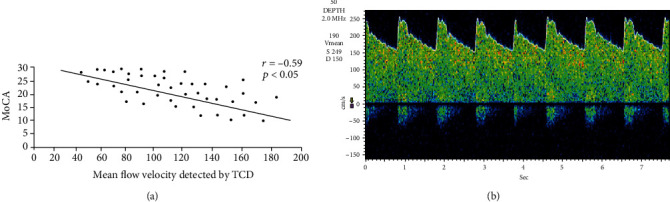
Correlation of mean flow velocity and MoCA.

Spearman correlation showed inverse linear correlation between the total MoCA and the average MFV (mean of all the arteries) (*r* = −0.59, *p* < 0.05) (Figure 3(a)).

### 3.2. Patients' Demographics and Risk Factors (Table 2)

The poor cognitive function group was older compared with the good cognitive function group (56.14 ± 15.27 years versus 49.15 ± 12.93 years, *p* < 0.01), with no significant difference regarding the sex between the groups (*p* = 0.42).

Cases with impaired cognition in comparison with cases with intact cognitive functions showed significantly higher mean blood glucose level (181.64 ± 34.42  mg% versus 144.83 ± 18.32  mg%, *p* = 0.03), significantly higher mean SBP (163.32 ± 19.43  mmHg versus 144.47 ± 17.38  mmHg, *p* = 0.02), and significantly higher mean DBP (104.65 ± 8.99  mmHg versus 91.47 ± 5.87  mmHg, *p* = 0.005), while cholesterol, triglyceride, and smoking were not significantly different between both groups (*p* = 0.32, *p* = 0.42, and *p* = 0.28, respectively).

### 3.3. Clinical and Radiological Scales (Table 2)

Cases with impaired cognition in comparison with cases with intact cognitive functions showed significantly lower mean GCS (9.36 ± 3.76 versus 13.16 ± 1.78, *p* = 0.01), significantly higher mean modified Fisher scale grades (*p* = 0.03), and significantly higher mean Hunt and Hess scale grades (*p* = 0.01).

Our results showed that the total cases complicated by hydrocephalus in the acute phase were 7 patients (17.5%) and the cases with impairment of the cognitive functions were accompanied by a higher incidence of hydrocephalus compared with patients with intact cognitive functions (44.4% versus 9.7%, *p* = 0.01).

Our study showed that the total cases complicated by DCI in the acute phase were 12 patients (30%) and the cases with impairment of the cognitive functions were accompanied by a higher incidence of DCI compared with patients with intact cognitive functions (77.8% versus 16.1%, *p* < 0.001).

### 3.4. TCD Parameters among Studied Patients (Tables 3 and 4)

Table 3 shows the TCD results in cases with cognitive impaired functions and cases with intact cognitive functions in the anterior, middle, and posterior cerebral arteries on both right and left sides during the 1st, 3rd, 5th, 7th, and 10th days from the onset of SAH.

Spearman correlation showed a significant correlation linking MFV of cerebral arteries, and the occurrence of impairment of the cognition (MoCA) was demonstrated in the following cerebral arteries: MCA LT 3, MCA LT 5, MCA LT 7, ACA LT 5, PCA LT 3, PCA LT 5, PCA LT 7, and MCA Rt 5, MCA Rt 10, ACA RT 5, and PCA RT 3 (Table 4).

**Figure 4:**
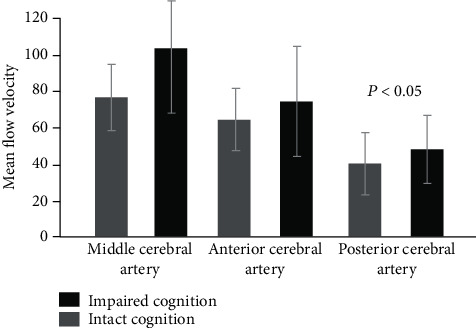
The average MFVs of the cerebral arteries measured within the first 10 days in aSAH patients with cognitive impairment and aSAH patients with intact cognition.

MFVs estimated during the first ten days in cases with cognitive impairment (MCA = 100.57 ± 29.87  cm/s, ACA = 72.47 ± 15.23  cm/s, and PCA = 50.73 ± 5.89  cm/s) were significantly higher than MFVs estimated in cases with intact cognitive functions (MCA = 78.65 ± 13.27  cm/s, ACA = 66.73 ± 9.48  cm/s, and PCA = 45.76 ± 5.64  cm/s) and *p* < 0.05 for all vessels (Figure 4).

### 3.5. Prediction of Impairment of Cognition (Table 5)

**Figure 5:**
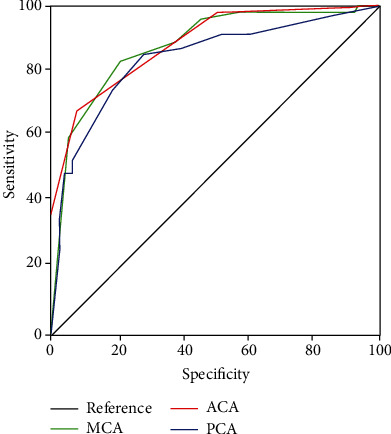
MFV cut-off values detected by the ROC curve.

Table 5 shows that the MFV cut-off values detected for the anticipation of impairment of cognition accomplished a sensitivity and specificity more than 70%. In the MCA, the estimated cut-off value for MFV was equal to 86 cm/s; in the ACA, the cut-off value for MFV was equal to 68 cm/s, while in the PCA, the cut-off point for MFV was equal to 45 cm/s. The execution of the cut-off values was detected by the ROC curve (Figure 5).

Finally, logistic regression analysis detected that MFV ≥ 86  cm/s in the MCA is significantly accompanied by a fourfold increased chance of impaired cognition (OR 4.15, 95% CI 2.09-12.35, *p* < 0.01), while MFV ≥ 68  cm/s in the ACA is significantly accompanied by a two-fold increased chance of impaired cognition (OR 2.21, 95% CI 1.41–8.57, *p* < 0.01), and MFV ≥ 45  cm/s in the PCA is significantly accompanied by a twofold increased chance of impaired cognition (OR 2.09, 95% CI 1.11–6.73, *p* < 0.05) (Table 5).

## 4. Discussion

In our study, 22.5% of patients had impaired cognitive functions after aSAH in the follow-up after 3 months, and 77.5% of aSAH patients had intact cognition with only mild cognitive dysfunction in memory. Hydrocephalus, CV, DCI, and increased MFV were highly associated with poor cognitive functions.

### 4.1. Cognitive Function in aSAH

Aneurysmal subarachnoid hemorrhage is a critical cause of short-term or long-term impaired cognition of variable degrees, with a frequency ranged from 7% up to 60% [34]. Researchers have detected that aSAH leads to brain damage and impaired cognition caused by several factors, which incorporate DCI, direct injury by the hemorrhage itself, elevated intracranial pressure, and hydrocephalus [35].

In our study, the patients with impaired cognitive function had a significant reduction of the total cognitive impairment estimated by the total MoCA score compared to patients with intact cognition (23.89 ± 2.46 versus 27.12 ± 2.78, *p* < 0.001). Typically similar to past studies on aSAH in the chronic stages, which has demonstrated that memory is the most common and the most frequent cognitive dysfunction [36, 37], and the impaired memory was improved over time [38, 39]. Our study showed significant impairment in the patients with cognitive impairment in the subtests of MoCA especially the domains of memory, executive functions, naming, and attention. While in the domains of the visual-spatial ability, language and orientation were nonsignificantly diminished in cases of SAH with impaired cognition.

#### 4.2. Risk Factors and Clinical Scales in aSAH Patients with Cognitive Impairment

The poor cognitive function group was of older age compared with the good cognitive function group with no important difference in the sex distribution in both groups and showed significantly higher mean blood glucose levels and significantly higher mean systolic and diastolic BP, while cholesterol, triglyceride, and smoking were not significantly differed between both groups. Cerebral arteries are the principal target of the harmful impacts of hypertension on the brain [40], resulting in cerebrovascular changes and neuropathological changes causing the cognitive dysfunctions [41, 42]. Hypertension is the prominent risk factor for the cerebrovascular damage, and the marked decrease in stroke morbidity and mortality has been credited to the treatment of hypertension [43, 44].

The predominance of impaired cognitive functions is especially higher in diabetic patients [45] and older patients [36], as they showed macrovascular and microvascular disorders that were related to the cognitive impairment in these patients [45].

Our study detected that cases with impaired cognition in comparison with cases with intact cognition showed significantly lower mean GCS, higher mean modified Fisher scale grades, and significantly higher mean Hunt and Hess scale grades. Similarly, the results of Shen and his colleague (2018) showed that the patients with GCS score of ≤7 at the onset, Hunt and Hess grade of ≥3, and Fisher grade of ≥3 were more likely to develop impaired cognition [46].

#### 4.3. Cerebral Vasospasm

Patients with impaired cognitive functions were accompanied by a more incidence of CV compared with patients with intact cognitive functions. CV is a serious complication of aSAH, possibly leading to delayed ischemia and focal neurological deficits and associated with cognitive dysfunction [47–49], and the improvement of the treatment of cerebral vasospasm has led to studies not finding any associations [50, 51]. CV occurred in 26 (65%) of patients with aSAH in our study, but the proper medication and the continuous monitoring of cases and early diagnosis of CV prevent the development of irreversible DCI. So, CV was not associated with a statistically significant incidence of cognitive impairment.

#### 4.4. Effects of Cerebral Ischemia and Hydrocephalus on Cognition

Acute hydrocephalus and recent cerebral ischemia associated with increased intracranial pressure after the bleeding, causing microvascular damage and impairment of the autoregulation leading to impaired cognition in the acute stage of aSAH, were demonstrated in the study of Kreiter and colleagues [36]. This link is clarified by proposing that cerebral edema may be caused by transient cerebral ischemia. In our study, the total cases complicated by hydrocephalus in the acute phase were 7 patients (17.5%) and patients with impaired cognitive functions were accompanied by a higher incidence of hydrocephalus in comparison with cases with intact cognition. Also, Chen and his colleagues (2017) found that hydrocephalus occurred in 20% of cases in the early phase (within 2 weeks) of aSAH, while chronic hydrocephalus occurred in about 10%-20% of cases later phase of aSAH (after 2 weeks) [52]. Acute hydrocephalus in patients with aSAH was associated with cognitive deficits, compared with patients not complicated by hydrocephalus [53]. Chronic hydrocephalus and impaired cognitive functions are late complications that occur after the initial subarachnoid hemorrhage [54]. It is due to dividing within the arachnoid space, which prevents the reabsorption of CSF and leads to dilatation of the ventricular system [55].

#### 4.5. Delayed Cerebral Infarction (DCI) and CI

Our study showed that the patients with impaired cognitive functions were accompanied by a higher incidence of DCI in comparison with cases with intact cognitive functions (77.8% versus 16.1%, *p* < 0.001). Also, Chu and his colleagues found that cases with DCI have more frequent impairment of the cognition. The most common affected domains of cognitive functions in aSAH cases with DCI were memory, language, and skill domains. These affected domains were rare in aSAH cases in the absence of DCI [56]. Similarly, Eagles et al. (2019) concluded that the development of DCI was a strong predictor of impaired cognition after aSAH [57]. This recommends the proposal that DCI may be considered as a therapeutic target for neuroprotection following aSAH [23].

#### 4.6. TCD and Cognitive Impairment

Our study detected a significant correlation linking MFV of cerebral arteries and the cognitive impairment, and the Spearman correlation showed an inverse linear relationship linking the total MoCA and the MFV of cerebral arteries. MFV ≥ 86  cm/s in the MCA is significantly accompanied by a fourfold elevated chance of impaired cognition, MFV ≥ 68  cm/s in the ACA is significantly accompanied by a twofold elevated chance of impaired cognition, and MFV ≥ 45  cm/s in the PCA is significantly accompanied by a twofold elevated chance of impaired cognition.

TCD assessment gives a clear physical demonstration of the impairment of brain perfusion and provides a suitable, noninvasive tool to evaluate the effectiveness of the medical treatments on cerebral blood flow or uncover early impairment of the cognition [58] and assessing the impact of medical interventions on cerebral blood flow [59]. TCD is especially beneficial in individuals with impaired cognition, because it can be utilized in older patients with metal inserts or pacemakers, and unlike CT or MRI scans can be transferred to patients in ICU [60].

TCD is able to evaluate the cerebral hemodynamics, the arterial perfusion, and the intracranial small vessel compliance [61–63] and gives useful indices of the incidence and severity of small vessel disease and executive dysfunction. The changes detected by TCD in vascular cognitive impairment showed a global distribution of cerebral hypoperfusion associated with an increase in the vascular resistance that may be due to microcirculation pathology and accompanied by small vessel and capillary damage [64]. Hypoperfusion might cause ischemic damage of white matter tracts and interrupt the subcortical tracts, so causing the various cognitive symptoms and especially impaired executive function [65–67].

#### 4.7. Limitations

This work represents a small-sized sample of SAH. The intermittent recording TCD may miss episodes of cerebral vasospasm. The techniques of TCD are highly operator dependent and significantly limit its clinical utility [68, 69]. So, TCD must be carried out by experienced operators to ensure proper and consistent recordings from the cerebral vessels and through the proper ultrasonography windows. One of the limitations is the difficulties in recruitment of age-matched healthy controls without any imaging evidence of subcortical ischemic vascular disease. Another limitation in our study is inability to insonate the cerebral vessels in some patients. Similarly, the literatures reported that 5–20% of patients will have difficult views causing uninterpretable transcranial windows and recordings [70, 71]. A significant limitation of TCD is that it does not give direct anatomical information about cerebral vessels. Moreover, the spatial resolution of TCD is limited for ACA and PCA and the diagnostic accuracy of TCD for detection of ACA and PCA vasospasm is limited [72, 73].

## 5. Conclusion

22.5% of patients had cognitive impairment after aSAH in the follow-up after 3 months. Acute hydrocephalus and delayed cerebral ischemia are the powerful predictors of impaired cognition after aSAH. TCD detected an inverse linear correlation between the average MFV and the total MoCA. MFV ≥ 86  cm/s in the MCA is significantly accompanied by a fourfold increased risk of impaired cognition, MFV ≥ 68  cm/s in the ACA is significantly accompanied by a twofold increased risk of impaired cognition, and MFV ≥ 45  cm/s in the PCA is significantly accompanied by a twofold increased risk of impaired cognition.

## Figures and Tables

**Table 1:** Classification of patients of SAH according to cognitive impairment.

	Good cognitive functions	Poor cognitive functions	*p* value
Number (%)	31 (77.5%)	9 (22.5%)	
MoCA test scores			
Visual-spatial ability	3.67 ± 1.18	3.41 ± 1.19	*p* = 0.39
Naming	2.51 ± 0.47	2.21 ± 0.39	*p* < 0.05^∗^
Executive functions	3.39 ± 0.61	2.62 ± 1.31	*p* < 0.05^∗^
Attention	4.28 ± 0.71	3.81 ± 1.35	*p* = 0.009^∗^
Language	2.59 ± 0.34	2.39 ± 0.49	*p* = 0.07
Memory	4.29 ± 0.58	3.57 ± 1.08	*p* < 0.005^∗^
Orientation	5.24 ± 0.62	4.89 ± 0.87	*p* = 0.09
Total MoCA score	27.12 ± 2.78	23.89 ± 2.46	*p* < 0.001^∗^

**Table 2:** Demographics, risk factors, and initial clinical scales at the onset.

	Intact cognition group (*n* = 31)	Impaired cognition group (*n* = 9)	*p* value
Number (%)	31 (77.5%)	9 (22.5%)	
Age (year)	49.15 ± 12.93	56.14 ± 15.27	*p* < 0.01^∗^
Sex F/M	16/15	6/3	*p* = 0.42
Glycemia (mg%)	144.83 ± 18.32	181.64 ± 34.42	*p* = 0.03^∗^
Cholesterol (mg%)	161.94 ± 18.94	172.47 ± 26.62	*p* = 0.32
Triglyceride (mg%)	151.29 ± 27.36	166.35 ± 29.37	*p* = 0.42
Smoking	11 (35.5%)	5 (55.6%)	*p* = 0.28
SBP (mmHg)	144.47 ± 17.38	163.32 ± 19.43	*p* = 0.02^∗^
DBP (mmHg)	91.47 ± 5.87	104.65 ± 8.99	*p* = 0.005^∗^
GCS	13.16 ± 1.78	9.36 ± 3.76	*p* = 0.01^∗^
Modified Fisher scale grades			
2	15 (48.4%)	2 (22.2%)	*p* = 0.03^∗^
3	12 (38.7%)	2 (22.2%)	
4	4 (12.0%)	5 (55.6%)	
Hunt and Hess scale grades			
1	14 (45.2%)	1 (11.1%)	*p* = 0.01^∗^
2	9 (29%)	1 (11.1%)	
3	8 (25.8%)	7 (66.7%)	
Hydrocephalus (total 7 patients = 17.5%)			
Present	3 (9.7%)	4 (44.4%)	*p* = 0.01^∗^
Absent	28 (90.3%)	5 (554.6%)	
Cerebral vasospasm (total 26 patients = 65%)			
Present	18 (58.1%)	8 (88.8%)	*p* = 0.08
Absent	13 (41.9%)	1 (11.2%)	
Delayed cerebral ischemia (total 12 patients = 30%)			
Present	5 (16.1%)	7 (77.8%)	*p* < 0.001^∗^
Absent	27 (83.9%)	2 (22.2%)	

SBP: systolic blood pressure; DBP: diastolic blood pressure; DM: diabetes mellitus.

**Table 3:** TCD parameters (MFV cm/s) in patients with intact cognition and patients with impaired cognition.

	TCD left side			TCD right side		
	Intact cognition group (*n* = 31)	Impaired cognition group (*n* = 9)	*p*	Intact cognition group (*n* = 31)	Impaired cognition group (*n* = 9)	*p*
MCA 1	69.98 ± 11.54	77.34 ± 10.17	0.215	66.95 ± 11.25	73.58 ± 12.65	0.293
MCA 3	71.36 ± 12.97	87.76 ± 16.94	**0.043**	71.96 ± 11.95	79.05 ± 16.58	0.253
MCA 5	76.26 ± 19.37	105.92 ± 41.13	**0.021**	74.56 ± 17.34	97.69 ± 40.76	**0.032**
MCA 7	84.48 ± 18.37	136.45 ± 55.73	**0.015**	81.36 ± 11.86	82.52 ± 10.47	0.841
MCA10	86.95 ± 14.50	88.16 ± 15.50	0.589	87.54 ± 17.78	128.91 ± 18.69	**0.031**
ACA 1	55.65 ± 8.57	59.42 ± 10.21	0.754	56.87 ± 5.86	57.85 ± 9.46	0.642
ACA 3	62.68 ± 9.34	63.13 ± 14.34	0.685	61.72 ± 9.72	63.34 ± 14.65	0.633
ACA 5	62.96 ± 10.45	73.45 ± 23.79	**0.036**	63.46 ± 7.68	70.96 ± 23.12	**0.038**
ACA 7	65.56 ± 11.37	70.87 ± 21.54	0.114	62.85 ± 7.98	63.32 ± 9.75	0.883
ACA10	66.95 ± 10.38	69.56 ± 20.32	0.121	64.68 ± 9.13	69.76 ± 19.48	0.153
PCA 1	39.25 ± 2.78	40.57 ± 5.67	0.573	40.68 ± 2.55	40.96 ± 5.94	0.865
PCA 3	40.68 ± 2.56	45.96 ± 5.57	**0.032**	41.32 ± 4.62	45.96 ± 4.58	**0.041**
PCA 5	42.82 ± 5.46	50.86 ± 5.71	**0.011**	44.25 ± 2.78	48.28 ± 10.13	0.162
PCA 7	44.25 ± 4.96	67.94 ± 19.65	**0.021**	44.97 ± 2.78	49.61 ± 5.37	0.059
PCA 10	47.57 ± 5.35	47.82 ± 5.35	0.935	47.89 ± 4.63	49.36 ± 5.21	0.863

ACA: anterior cerebral artery; MCA: middle cerebral artery; PCA: posterior cerebral artery.

**Table 4:** Correlation between TCD findings and cognitive impairment (MoCA).

	TCD left side		TCD right side	
	*r*	*p* value	*r*	*p* value
MCA 1	-0.289	0.225	-0.264	0.265
MCA 3	-0.471	**0.035**	-0.267	0.261
MCA 5	-0.517	**0.021**	-0.483	**0.034**
MCA 7	-0.591	**0.012**	0.075	0.825
MCA 10	-0.179	0.586	-0.489	**0.032**
ACA 1	0.067	0.843	0.076	0.747
ACA 3	0.085	0.765	0.114	0.643
ACA 5	-0.472	**0.035**	-0.468	**0.039**
ACA 7	-0.164	0.625	0.013	0.976
ACA 10	-0.346	0.092	-0.362	0.178
PCA 1	-0.148	0.577	0.039	0.897
PCA 3	-0.489	**0.032**	-0.464	**0.041**
PCA 5	-0.604	**0.011**	-0.312	0.119
PCA 7	-0.518	**0.021**	-0.341	0.091
PCA 10	0.012	0.981	0.074	0.758

ACA: anterior cerebral artery; MCA: middle cerebral artery; PCA: posterior cerebral artery.

**Table 5:** Prediction of the cognitive impairment and binary logistic regression for MFV cut-off values measured by TCD.

	MCA	ACA	PCA
MFV cut-off	≥86	≥68	≥45
Sensitivity	0.76	0.71	0.69
Specificity	0.81	0.74	0.70
PPV	0.86	0.81	0.77
NPV	0.74	0.72	0.69
OR	4.15	2.21	2.09
95% CI	2.09-12.35	1.41–8.57	1.11–6.73
*p* value	*p* < 0.01	*p* < 0.01	*p* < 0.05

## Data Availability

The datasets generated and analyzed during the current study are not publicly available due to institutional limitations, yet they are available from the corresponding author on reasonable request.
